# Impact of Preoperative Neutrophil to Lymphocyte Ratio and Postoperative Infectious Complications on Survival After Curative Gastrectomy for Gastric Cancer: A Single Institutional Cohort Study

**DOI:** 10.1097/MD.0000000000003125

**Published:** 2016-03-18

**Authors:** Yasuhiko Mohri, Koji Tanaka, Yuji Toiyama, Masaki Ohi, Hiromi Yasuda, Yasuhiro Inoue, Masato Kusunoki

**Affiliations:** From the Department of Gastrointestinal and Pediatric Surgery, Mie University Graduate School of Medicine, Tsu, Mie, Japan.

## Abstract

Although postoperative complications are associated with a poor long-term prognosis after resection of several solid tumors via an undetermined mechanism, there are few related reports in gastric cancer patients. Preoperative elevated neutrophil to lymphocyte ratio (NLR) reflects a systemic inflammatory response and is a predictor of poor survival in gastric cancer. The relationship between preoperative NLR and postoperative complications and the impact of these 2 factors on survival in gastric cancer remains unclear. Our aim is to examine the association between postoperative complications and survival, and preoperative NLR in patients undergoing curative resection for gastric cancer.

We enrolled a total of 404 consecutive patients with gastric cancer undergoing curative gastrectomy between January 1, 2000 and December 31, 2011. Multivariable analyses were performed to correlate preoperative and operative variables with postoperative complications and to correlate complications with long-term survival after gastrectomy.

Postoperative infectious and noninfectious complication rates were 17.6% and 7.9%, respectively. Preoperative NLR independently predicted the development of postoperative infectious complication, but not the development of postoperative noninfectious complications after gastrectomy. Both elevated NLR and postoperative infectious complication were independently associated with long-term survival. Also, patients with both elevated NLR and the development of postoperative infectious complication had the worst long-term survival.

NLR independently predicted the development of postoperative infectious complication and lower survival after gastrectomy. Elevated NLR could trigger postoperative infectious complication and increase the risk of recurrence in patients with postoperative infectious complication after gastrectomy.

## INTRODUCTION

Gastric cancer is the 4th most common malignancy and 2nd most common cause of cancer-related death worldwide.^1^ Early detection and surgical resection of gastric cancer confers the greatest chance of long-term survival. Despite improvements in surgery and perioperative care resulting in reduced mortality for gastric cancer resections, postoperative morbidity remains a clinically significant problem with a reported range of 20% to 46%.^2–4^ Postoperative morbidity is associated not only with increased length of hospitalization and greater economic cost but also with poor long-term survival in several solid tumors.^5–7^ To date, however, there are few studies suggesting postoperative morbidity is associated with poor overall and cancer-specific survival after gastrectomy for gastric cancer.^8–10^ Although the mechanism linking postoperative morbidity with poor long-term prognosis after cancer resection is not known, reducing postoperative morbidities may improve long-term survival.

The preoperative neutrophil to lymphocyte ratio (NLR), a marker for the systemic inflammatory response, independently predicts poor prognosis after resection of a range of malignant neoplasms,^11^ including gastric cancer.^12^ Evidence suggests that NLR has a direct role in promoting aggressive tumor phenotypes and disease recurrence. Recently, a link between systemic inflammation and postoperative morbidity was identified by Moyes et al.^13^ In their study, the presence of the preoperative systemic inflammatory response itself independently predicted postoperative infectious complications in patients undergoing curative resection for colorectal cancer. These data suggest that complex links exist between systemic inflammation, postoperative morbidity, and long-term survival in patients with colorectal cancer. However, the relationship between NLR and postoperative complications remains uncertain and we aimed to further define these links in patients with gastric cancer. First, we examined the association between the preoperative NLR and postoperative morbidities in patients undergoing gastrectomy for gastric cancer. Second, we defined the prognostic value of postoperative morbidities in this context using multivariable analyses that included NLR.

## METHODS

### Ethics Statements

The study was approved by the Mie University Hospital ethics committee and was conducted in accordance with the guidelines of the 1975 Declaration of Helsinki. The need for informed patient consent was waived because of the retrospective nature of the study.

### Patients

Patients undergoing curative gastrectomy for gastric cancer between January 1, 2000 and December 31, 2011 at a single institution (Department of Gastrointestinal Surgery, Mie University Hospital) were identified using a prospectively maintained database. These data were analyzed retrospectively, with additional information obtained from hospital records. Preoperative staging comprised computed tomography of the chest, abdomen, and pelvis; upper gastrointestinal contrast radiographic series; and esophagogastroduodenal endoscopy. Clinicopathologic variables were recorded for each patient. Laboratory blood test data collected within 2 weeks before surgery included preoperative neutrophil and lymphocyte count, and carcinoembryonic antigen and cancer antigen 19-9 levels. Resected specimens were examined histopathologically and staged according to the International Union Against Cancer tumor-node-metastasis (TNM) classification.^14^ No patient had clinical evidence of infection or other inflammatory conditions at the time of sampling.

### Outcome Measure

The main short-term postoperative outcome measures were mortality and morbidity, with morbidity including infectious complications and noninfectious complications. Morbidity and mortality were defined as occurring within 30 days of surgery. Details of the complications were obtained from the prospectively collated database and, where necessary, from the medical records. Complications were further categorized into either: infectious or noninfectious complications. Recorded infectious complications included wound infection (superficial or deep infection that required treatment with antibiotic agents or wound drainage); intra-abdominal abscess (intra-abdominal fluid collection associated with fever or leukocytosis that discharged spontaneously or required surgical or radiologically guided drainage, with positive blood or fluid culture); and respiratory tract infection (respiratory symptoms and signs and infiltrate on chest radiography associated with fever or leukocytosis requiring antibiotic drug treatment). Sepsis was defined as the presence of 2 or more systemic inflammatory response syndrome criteria.^15^ Noninfectious complications consisted of small bowel obstruction, anastomotic stricture, postoperative hemorrhage including intraluminal bleeding at any surgical site, cardiac complications, pneumothorax, deep vein thrombosis, liver dysfunction, and renal failure.

### Follow-Up

The follow-up protocol included clinical review 6 weeks after hospital discharge, followed by clinical evaluation, serum investigations (including carcinoembryonic antigen and cancer antigen 19-9 measurement), and abdominal ultrasonography, all performed every 3 months for the 1st year, every 6 months for the 2nd year, and then annually until 5 years after gastrectomy. Abnormal results during surveillance triggered further investigation. The long-term outcome measures were overall and cancer-specific survival (as of December 2014). Patients who died within 30 days of surgery were excluded from overall and cancer-specific survival analyses and patients who died from causes other than gastric cancer recurrence were excluded from the cancer-specific survival analysis.

### Statistical Analysis

We determined the optimal discriminator value for NLR using receiver operating characteristic (ROC) curve analysis. At each value, the sensitivity and specificity for each outcome under study were plotted, generating an ROC curve. The ratio closest to the point with maximum sensitivity and specificity was selected as the cutoff value and comparisons between groups of patients were performed using the chi-squared test, as appropriate. Univariate and multivariate logistic regression analyses were used to examine the effect of variables on the development of postoperative infectious and noninfectious complications. Univariate associations with long-term survival were determined using univariate Cox regression analysis, Kaplan–Meier analysis, and the log-rank test. Multivariable analyses were performed using Cox proportional hazards regression analysis (using a stepwise backward procedure), incorporating all variables with *P* < 0.10 on univariate analysis. Statistical significance was defined as *P* < 0.05. All analyses were conducted using the software program, SPSS version 16.0 (SPSS Inc., Chicago, IL).

## RESULTS

An ROC curve analysis was performed for postoperative morbidity and death from any cause. For all 404 gastric cancer patients, an NLR of 3.0 had the highest sensitivity and specificity for both outcomes. Thereafter, patients were stratified into 2 groups: low NLR (≤3) and high NLR (>3). The relationship between the preoperative NLR and clinicopathological characteristics in patients (n = 404) who underwent curative surgery for gastric cancer is shown in Table 1. There were 283 men (70%) and 121 women (30%); the median patient age at the time of surgery was 67.0 years (range, 30–88 years); and tumor size ranged from 0.4 to 15.0 cm, with a median size of 3.0 cm. The majority of patients presented with tumor located in the lower two-thirds of the stomach (77%) and TNM stage I disease (64%). One hundred eleven patients (27.5%) had a total gastrectomy and 290 patients (72.5%) underwent partial gastrectomy including distal or proximal gastrectomy. The majority of patients had low preoperative NLR (≤3.0) (80%). High NLR (>3) was significantly associated with older age, high American Society of Anesthesiologists score, histological differentiation, advanced TNM disease stage, and the development of postoperative morbidity.

**TABLE 1:**
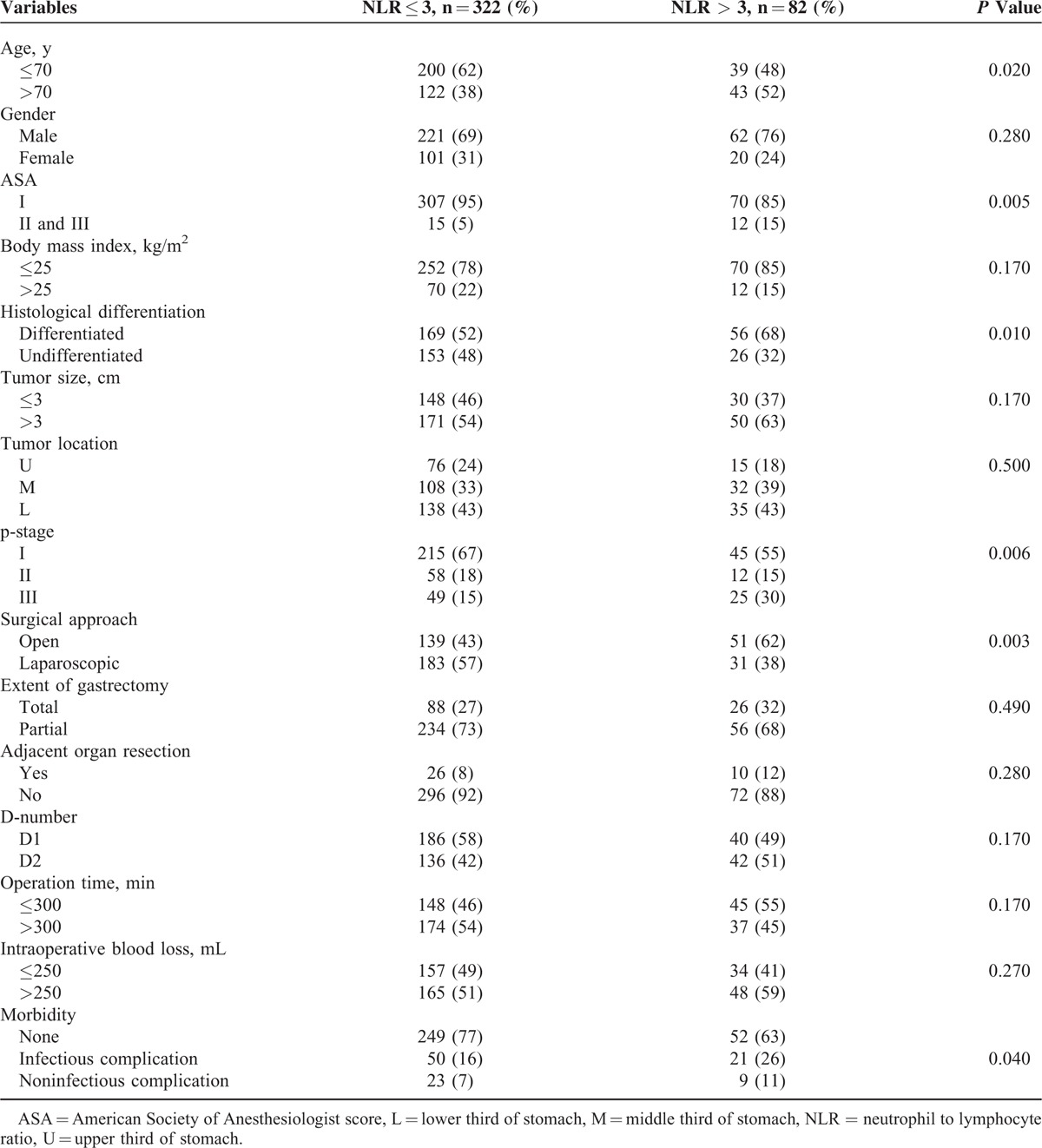
Relationship Between Preoperative Neutrophil to Lymphocyte Ratio and Clinicopathological Characteristics in Patients Undergoing Curative Surgery for Gastric Cancer (n = 404)

### Short-Term Outcomes

Postoperative morbidity occurred in 103 patients (25.5%) after gastrectomy, with a total of 119 events recorded (Table 2). Infectious complications were most common, developing in 71 patients (17.6 %) and noninfectious complications occurred in 32 patients (7.9%). Thirty-day mortality after gastrectomy was 0.5% (n = 2), with 1 patient developing infectious complication and another developing noninfectious complication.

**TABLE 2:**
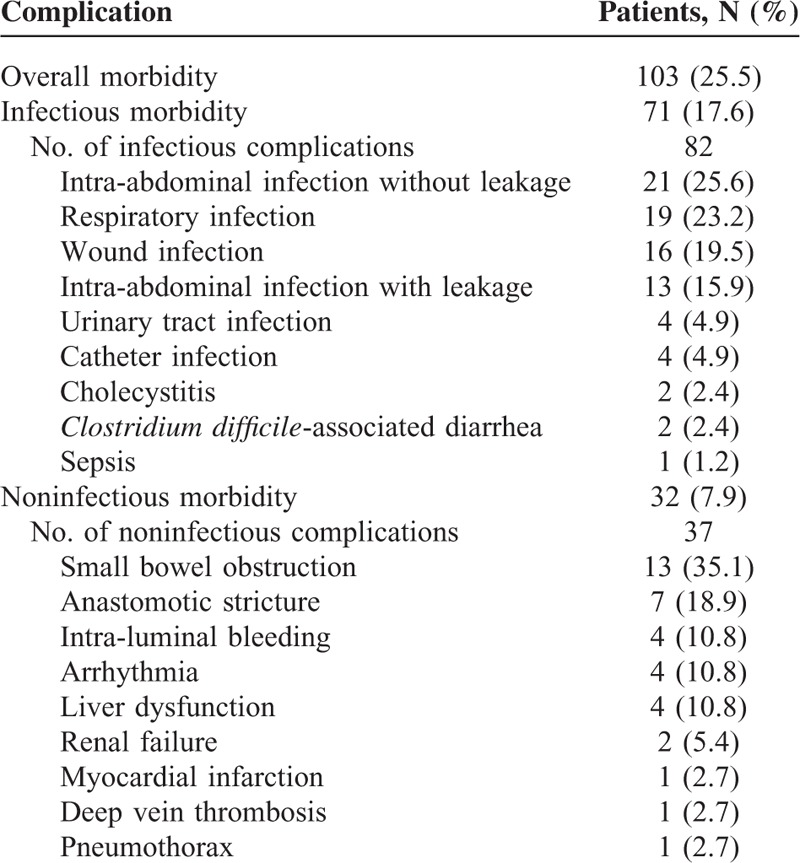
Complications After Gastrectomy for Gastric Cancer in 404 Patients

The results of univariate analysis for factors associated with postoperative infectious complications and noninfectious complications after gastrectomy are shown in Table 3. On univariate analysis, older age (*P* = 0.01), tumor location at upper third of stomach (*P* = 0.01), total gastrectomy (*P* = 0.02), adjacent organ resection (*P* = 0.03), and high NLR (*P* = 0.03) were significantly associated with postoperative infectious complication. Only total gastrectomy was significantly associated with noninfectious complication on univariate analysis.

**TABLE 3:**
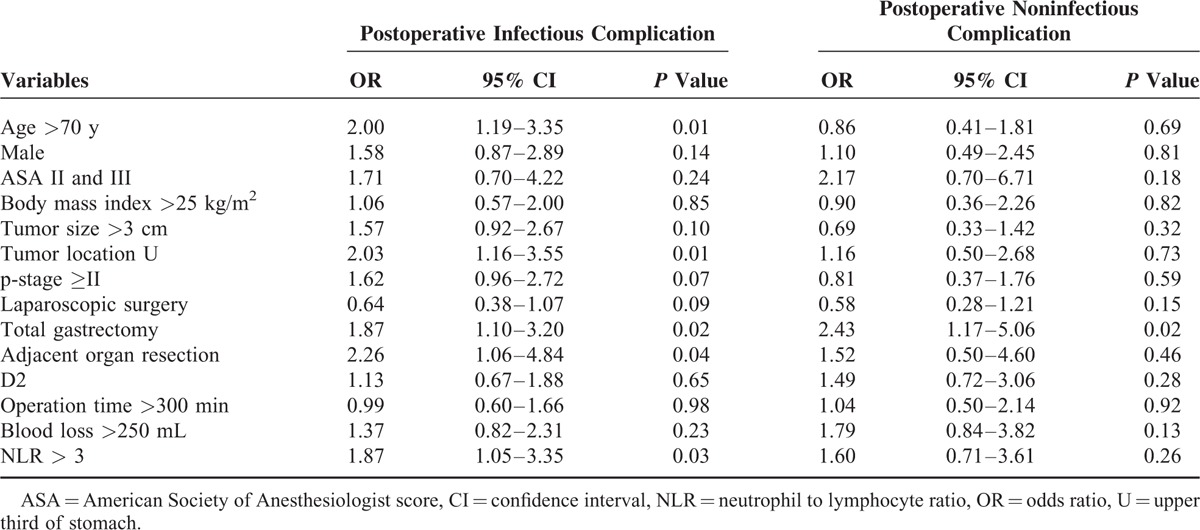
Clinicopathological Characteristics and Postoperative Morbidity in Patients Undergoing Gastrectomy: Univariate Logistic Regression Analysis

On multivariate analysis (Table 4), older age, tumor location at upper third of stomach, and elevated NLR were independent variables associated with the development of postoperative infectious complication.

**TABLE 4:**
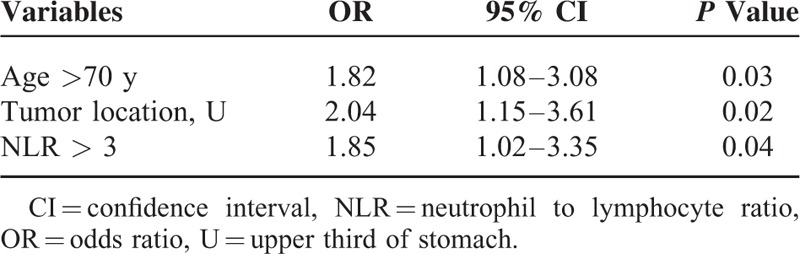
Multivariate Logistic Regression Analysis for Postoperative Infectious Complication

### Long-Term Outcomes

The overall median follow-up was 52.0 months. Three- and five-year overall survivals after gastrectomy were 85.6% and 79.8%, respectively. Three- and five-year cancer-specific survivals were 87.5% and 83.8%, respectively.

Univariate survival analyses were performed to determine the factors associated with overall survival and cancer-specific survival (Table 5). When variables (*P* < 0.1) from univariate analysis were entered into multivariable analyses (Table 6), both NLR and postoperative infectious complication were still independently associated with overall and cancer-specific survival after curative gastrectomy.

**TABLE 5:**
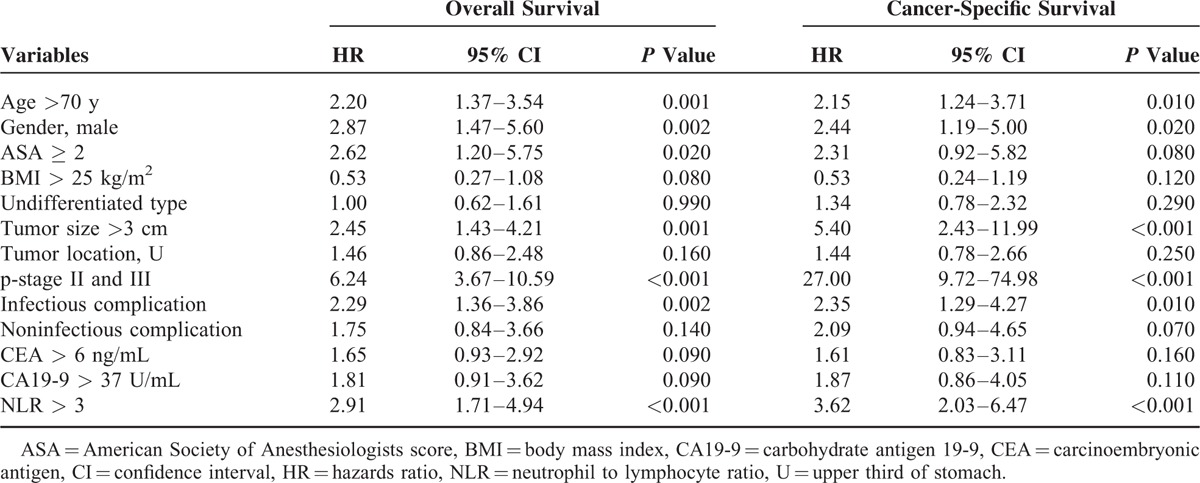
Univariate Cox Proportional Hazards Model for Overall and Cancer-Specific Survival

**TABLE 6:**
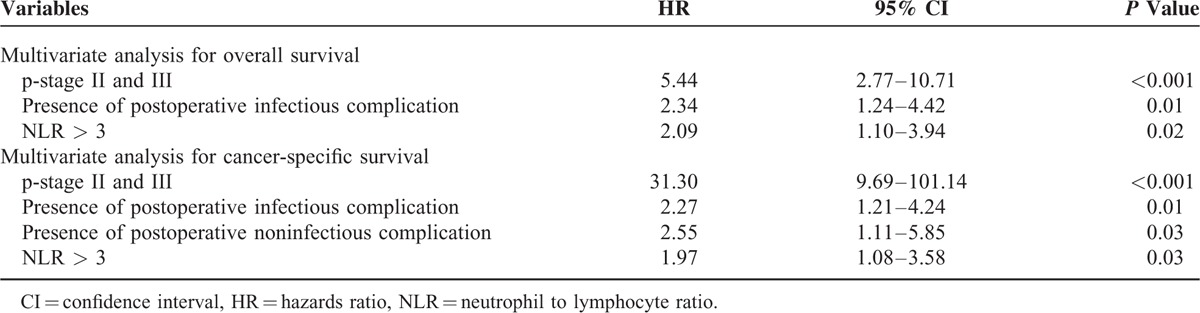
Multivariate Cox Proportional Hazards Model for Overall and Cancer-Specific Survival

**FIGURE 1:**
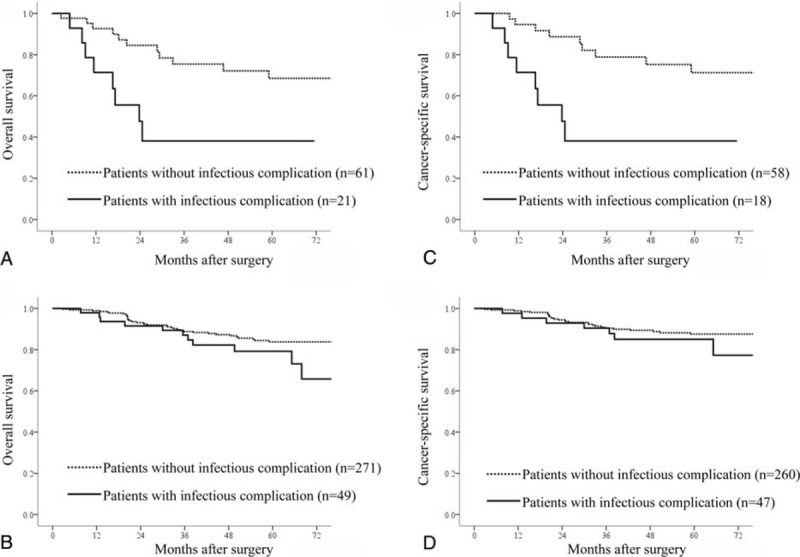
Comparison of overall and cancer-specific survival rates between patients with and without postoperative infectious complication. Solid line indicates the patients with postoperative infectious complication. Dotted line indicates the patients without postoperative infectious complication. (A) Relationship of overall survival in patients with high NLR (>3) (*P* = 0.01); (B) relationship of overall survival in patients with low NLR (≤3) (*P* = 0.06); (C) relationship of cancer-specific survival in patients with high NLR (>3) (*P* = 0.002); (D) relationship of cancer-specific survival in patients with low NLR (≤3) (*P* = 0.33).

We also examined the effect of NLR on survival in patients with or without the development of postoperative infectious complication. In patients with high NLR (>3), those with postoperative infectious complication had shorter overall (Figure 1A) and cancer-specific (Figure 1C) survival than those without postoperative infectious complication. However, in patients with low NLR (≤3), there was no significant difference in overall (Figure 1B) and cancer-specific (Figure 1D) survival between patients with and without postoperative infectious complication.

## DISCUSSION

Our results showed that preoperative NLR was independently associated with the development of postoperative infectious complication but not associated with the development of noninfectious complication. Also, both elevated NLR and the development of postoperative infectious complication were independently associated with overall and cancer-specific survival after curative resection for gastric cancer. In addition to increasing evidence suggesting that elevated NLR is related to key factors influencing survival in gastric cancer patients, our results demonstrated a significant relationship between NLR and short- and long-term outcomes and elevated NLR as a key factor in the relationship between postoperative infectious complication and poor survival in patients undergoing curative gastrectomy.

Several studies have demonstrated a correlation between systemic inflammation and complications after oncologic surgery.^11,14^ One study showed that the Glasgow Prognostic Score (a composite score derived from the levels of serum C-reactive protein level and albumin) and the total leukocyte count independently predicted infectious but not noninfectious complications after primary colorectal cancer resection.^11^ Another showed that neutrophil count independently predicted infectious complications after hepatectomy originating from colorectal cancer.^16^ We demonstrated that elevated NLR was independently associated with the development of postoperative infectious complication, but not associated with the development of postoperative noninfectious complications. This correlation was independent of upper tumor location, adjacent organ resection, and older age, which were also independent predictors of postoperative infectious complications and well-established as adverse short-term outcomes after curative gastrectomy.^17,18^

The association between elevated NLR and the development of postoperative infectious complication is likely complex and remains unclear; however, possible explanations exist. NLR, which is derived from the absolute neutrophil and absolute lymphocyte counts of a full blood count, is a routinely available marker of the systemic inflammatory response. The antibacterial responses of natural killer cells and activated T cells may be suppressed by increased number of neutrophils near the site of bacterial contamination.^19^ A high NLR reflects both a heightened neutrophil-dependent inflammatory response and a decreased lymphocyte-mediated antibacterial immune reaction, which may weaken the lymphocyte-mediated antibacterial cellular immune response and contribute to increasing bacterial invasion and growth. Also, circulating interleukin-6 level, which increases in various cancer patients including those with gastric cancer^20,21^ and which promotes the proliferation of immature neutrophils in the circulation and stimulates mature neutrophils to release superoxide anion^22^ as a reaction to surgical trauma, may contribute to oxygen radical-mediated tissue injury and bacterial invasion. Although the exact mechanism remains unclear, the results of our study suggest that NLR, as a simple preoperative inflammatory biomarker, may identify patients at increased risk for postoperative infectious complications after gastrectomy for gastric cancer.

Similar to findings in recent studies of gastric cancer resection, postoperative infectious complications were associated with shortened overall and cancer-specific survival after gastrectomy in our study.^8–10^ Major surgery elicits systemic inflammation and immunosuppression in patients.^23^ Postoperative complications may increase the magnitude and duration of this systemic inflammatory response, thereby predisposing to the growth of metastatic tumor cells and tumor recurrence.^24^ In particular, postoperative sepsis leads to an extended period of inflammation and immunosuppression^25^ that may contribute to metastatic proliferation.^26^ We also demonstrated that noninfectious complication was independently associated with cancer-specific survival but not with overall survival.

To our knowledge, there are no studies on the impact of noninfectious complication on survival in patients with gastric cancer. Although noninfectious complications also affect the postoperative systemic inflammatory response, further research is needed to determine the impact of noninfectious complication on long-term outcomes in gastric cancer surgery. Although it was previously reported that only the preoperative, and not the postoperative, systemic inflammatory response was associated with shortened cancer-specific survival after colorectal cancer resection,^27^ it remains possible that the specific molecular events involved or the magnitude of the systemic inflammation elicited regardless of complication types.

An alternative explanation for the link between infectious complications and cancer recurrence is that postoperative infectious complication is a manifestation of preexisting immune alteration that itself predisposes to recurrence. This is supported by the observation that elevated NLR, a known independent predictor of adverse long-term outcome,^11^ was independently associated with the development of postoperative infectious complications and by the results of based on our multivariable analyses. Although several studies^8–10^ found that postoperative infectious complications had independent prognostic significance after gastric cancer resection, none included inflammatory variables in the multivariable survival analyses. In our study, both postoperative infectious complications and elevated NLR independently predicted shortened overall and cancer-specific survivals after curative gastrectomy for gastric cancer. Also, although overall and cancer-specific survivals were significantly worse in patients that developed infection than in those without infection among patients with NLR > 3, survivals were not significantly different between patients with and without infectious complication among patients with NLR < 3. These data suggest that the prognostic value of postoperative complications is partially derived, to a significant degree, from their independent association with the preoperative systemic inflammatory response.

The mechanisms underlying the association between elevated NLR and poor survival of gastric cancer are poorly understood. One potential mechanism underlying the prognostic impact of NLR is that high NLR reflects an enhanced neutrophil response to tumors. Circulating neutrophils favor angiogenesis and proangiogenetic factor secretion including vascular endothelial growth factor; therefore, an elevated neutrophil count stimulates tumor angiogenesis and aids tumor progression.^28^ Another mechanism is that relatively low lymphocytes may weaken the lymphocyte mediated antitumor cellular immune response and some investigations have shown that increased neutrophils suppress the cytolytic activity of lymphocytes, natural killer cells, and activated T cells.^29^ Therefore, cancer cells in patients with elevated NLR may have a survival advantage resulting in poorer outcomes and increased recurrence.

Certain limitations in this study merit explanation. First, this was a retrospective study despite comprising a relatively large and consecutive data set. Second, patients who underwent an emergency operation, a resection margin (macro- or microscopic), or any type of neoadjuvant treatment were excluded, as these interventions can alter preoperative white blood cell population ratios.

## CONCLUSIONS

Our results suggest that the preoperative systemic inflammatory response, easily measurable before surgery, may be clinically useful to identify patients at increased risk for postoperative infectious complications and to predict long-term survival after gastrectomy. In addition to potentially guiding the allocation of existing treatment modalities, inflammatory variables themselves represent attractive therapeutic targets. Although it remains to be determined whether the preoperative systemic inflammatory response can be moderated, the development of novel therapeutic agents targeting varied aspects of the systemic inflammatory response raises the possibility that moderation of systemic inflammation may be used to improve perioperative outcomes and long-term prognosis after resection for cancer.

## References

[1] Jemal A Bray F Center MM Global cancer statistics. *CA Cancer J Clin* 2011; 61:69–90. 21296855 10.3322/caac.20107

[2] Sano T Sasako M Yamamoto S Gastric cancer surgery: morbidity and mortality results from a prospective randomized controlled trial comparing D2 and extended para-aortic lymphadenectomy—Japan Clinical Oncology Group Study 9501. *J Clin Oncol* 2004; 22:2267–2773. 15199090 10.1200/JCO.2004.10.184

[3] Cunningham D Allum WH Stenning SP Perioperative chemotherapy versus surgery alone for resectable gastroesophageal cancer. *N Engl J Med* 2006; 355:11–20. 16822992 10.1056/NEJMoa055531

[4] Papenfuss WA Kukar M Oxenberg J Morbidity and mortality associated with gastrectomy for gastric cancer. *Ann Surg Oncol* 2014; 21:3008–3014. 24700300 10.1245/s10434-014-3664-z

[5] Faird SG Aldouri A Morris-Stiff G Correlation between postoperative infective complications and long-term outcomes after hepatic resection for colorectal liver metastasis. *Ann Surg* 2010; 251:91–100. 19858702 10.1097/SLA.0b013e3181bfda3c

[6] Chauhan A House MG Pitt HA Postoperative morbidity results in decreased long-term survival after resection for hilar cholangiocarcinoma. *HPB (Oxford)* 2011; 13:139–147. 21241432 10.1111/j.1477-2574.2010.00262.x PMC3044349

[7] Law WL Choi HK Lee YM The impact of postoperative complications on long-term outcomes following curative resection for colorectal cancer. *Ann Surg Oncol* 2007; 14:2559–2566. 17522945 10.1245/s10434-007-9434-4

[8] Tsujimoto H Ichikura T Ono S Impact of infectious complications on long-term survival after potentially curative resection for gastric cancer. *Ann Surg Oncol* 2009; 16:311–318. 19037699 10.1245/s10434-008-0249-8

[9] Sierzega M Kolodziejczk P Kulig J Impact anastomotic leakage on long-term survival after total gastrectomy for carcinoma of the stomach. *Br J Surg* 2010; 97:1035–1042. 20632269 10.1002/bjs.7038

[10] Tokunaga M Tanizawa Y Bando E Poor survival rate in patients with postoperative intra-abdominal infectious complications following curative gastrectomy for gastric cancer. *Ann Surg Oncol* 2013; 20:1575–1583. 23076557 10.1245/s10434-012-2720-9

[11] Templeton AJ McNamara MG Šeruga B Prognostic role of neutrophil-to-lymphocyte ratio in solid tumors: a systematic review and meta-analysis. *J Natl Cancer Inst* 2014; 106:dju124. 24875653 10.1093/jnci/dju124

[12] Zhang X Zhang W Feng LJ Prognostic significance of neutrophil lymphocyte ratio in patients with gastric cancer: a meta-analysis. *PLoS ONE* 2014; 9:e111906. 25401500 10.1371/journal.pone.0111906 PMC4234250

[13] Moyes LH Leitch EF McKee RF Preoperative systemic inflammation predicts postoperative infectious complications in patients undergoing curative resection for colorectal cancer. *Br J Cancer* 2009; 100:1236–1239. 19319134 10.1038/sj.bjc.6604997 PMC2676538

[14] Sobin LH Gospodarowicz MK Witterkind CH International Union Against Cancer (UICC) TNM Classification of Malignant Tumors. 7th ed Oxford: Wiley-Blackwell; 2009.

[15] Bone RC Balk RA Cerra FB Definition for sepsis and organ failure and guidelines for the use of innovative therapies in sepsis. The ACCP/SCCM Consensus Conference Committee. American College of Chest Physicians/Society of Critical Care Medicine. *Chest* 1992; 136 (Suppl):e28. 10.1378/chest.101.6.1644 1303622

[16] Neal CP Mann CD Garcea G Preoperative systemic inflammation and infectious complications after resection of colorectal liver metastases. *Arch Surg* 2011; 146:471–478. 21502458 10.1001/archsurg.2011.50

[17] Kubota T Hiki N Nunobe S Significance of the inflammation-based Glasgow Prognostic Score for short- and long-term outcomes after curative resection of gastric cancer. *J Gastrointest Surg* 2012; 16:2037–2044. 23007284 10.1007/s11605-012-2036-x

[18] Kodera Y Sasako M Yamamoto S Identification of risk for the development of complications following extended and superextended lymphadenectomies for gastric cancer. *Br J Surg* 2005; 92:1103–1109. 16106493 10.1002/bjs.4979

[19] Shau H Kim A Suppression of lymphokine-activated killer induction by neutrophils. *J Immunol* 1988; 141:4395–4402. 3264311

[20] Kabir S Daar GA Serum levels interleukin-1, interleukin-6 and tumor-necrosis factor alpha in patients with gastric carcinoma. *Cancer Lett* 1995; 95:207–212. 7656232 10.1016/0304-3835(95)03895-4

[21] Ikuta S Miki C Tanaka K Serum immunosuppressive acidic protein as an interleukin-6 related index of deteriorating condition in gastric cancer patients. *Dig Surg* 2003; 20:532–538. 14534376 10.1159/000073700

[22] Djeu JY Serbousek D Blanchard DK Release of tumor necrosis factor by human polymorphonuclear leukocytes. *Blood* 1990; 76:1405–1409. 2207315

[23] Lundy J Ford CM Surgery, trauma and immune suppression: evolving the mechanism. *Ann Surg* 1983; 197:434–438. 6219640 10.1097/00000658-198304000-00010 PMC1352757

[24] Khuri SF Henderson WG DePalma RG Participants in the VA National Surgical Quality Improvement Program. Determinants of long-term survival after major surgery and the adverse effect of postoperative complications. *Ann Surg* 2005; 242:326–343. 16135919 10.1097/01.sla.0000179621.33268.83 PMC1357741

[25] Mynster T Christensen IJ Moesgaard F Effects of the combination of blood transfusion and postoperative infectious complications on prognosis after surgery for colorectal cancer. Danish RANX05 Colorectal Cancer Study Group. *Br J Surg* 2000; 87:1553–1562. 11091245 10.1046/j.1365-2168.2000.01570.x

[26] Panis Y Ribeiro J Chrétien Y Dormant liver metastases: an experimental study. *Br J Surg* 1992; 79:221–223. 1555087 10.1002/bjs.1800790309

[27] Crozier JE McKee RF McArdle CS Preoperative but not postoperative systemic inflammatory response correlates with survival in colorectal cancer. *Br J Surg* 2007; 94:1028–1032. 17437250 10.1002/bjs.5706

[28] Shamamian P Schwartz JD Pocock BJ Activation of progelatinase A (MMP-2) by neutrophil elastase, cathepsin G, and proteinase-3: a role for inflammatory cells in tumor invasion and angiogenesis. *J Cell Physiol* 2001; 189:197–206. 11598905 10.1002/jcp.10014

[29] Petrie HT Klassen LW Kay HD Inhibition of human cytotoxic T lymphocyte activity in vitro by autologous peripheral blood granulocytes. *J Immunol* 1985; 134:230–234. 3871101

